# Some Observations of the Uterine Sound

**Published:** 1886-07

**Authors:** William Warren Potter

**Affiliations:** 284 Franklin Street; Buffalo, N. Y.


THE BUFFALO MEDICAL AND SURGICAL JOURNAL

VOL. XXVI. JULY, 1886. NO. 12.

### Original Communications.

### SOME OBSERVATIONS ON THE UTERINE SOUND, WITH ESPECIAL REFERENCE TO ITS PLACE IN GYNÆCOLOGICAL THERAPEUTICS.

BY WILLIAM WARREN POTTER, M. D., Buffalo, N. Y.

'Twas but a few years ago, when the possession of a Fergusson's speculum, a caustic-holder, a uterine dressing forceps, and a Simpson's sound, was considered by the vast majority, an ample outfit for the practice of gynæcology. Some few, 'tis true, more favored than the many, added a number of other instruments to their armamentaria ; but these were, in the hands of the multitude which made pretense to practice the art, all-sufficient for the diagnosis, treatment, and cure (?) of all the sexual ills of woman.

The discovery by Sims of the speculum, which now, by almost universal consent, bears his name, and the consequent improvements in the methods of diagnosis which followed its introduction, soon led to the retirement of the round, or cylindrical, speculum of Fergusson, so that now it is almost unheard of, excepting as one of the curiosities that belong to a nearly obsolete method of practice. So, too, with the coming into vogue of the more exact methods of diagnosis and treatment of the Sims' school of gynæcology, the uterine port-caustique fell into comparative disuse; for it had now been found that many cases of acquired stenoses, as well as innumerable cicatricial bands, and other formations in the sexual tract, were produced by the indiscriminate, and all too general application of the nitrate of silver.

The uterine sound, however, has held sway for a much longer period, or to a much later day, and is only now, or recently, beginning to give way to other and safer, not to say better methods of diagnosis and treatment. It has been used in the treatment of almost every malady peculiar to the female pelvic organs, and I am very sure it is entirely within the range of truth to assert, that it has done woman more harm than any other instrument which has been employed in the management of her diseases. It is still, in the minds of many, a matter of some doubt as to just what its present status is, with reference to uterine therapeutics; and, on this account, I have ventured to lay the subject before the society at this time, hoping to elucidate, thereby, valuable opinons and experiences, which will contribute towards a better and more enlightened understanding of the question.

When Simpson, in 1843, published his first memoir on the uterine sound, it attracted the attention of the profession throughout the civilized world—at least those members of it more especially interested in, or giving attention to, the treatment of the diseases of women. Professor Simpson proposed the instrument, which bears his name, for the purpose of diagnosticating, or aiding in the diagnosis of, many of the diseases of women, which had before been either little understood, or greatly misunderstood, or else confounded with other maladies. In this he wrought a seven-league-boot stride in advance in his special field, and we are indebted to him for our first intelligent understanding of many of those diseases which now seem easily differentiated.

Indeed, previously to the knowledge which the sound afforded, though the retroverted uterine body had often been felt resting in the concavity of the sacrum, it had not been possible to recognize its true nature and import by the touch alone. It had been confounded with, or mistaken for, almost every variety of tumor or morbid growth which might, and often does, lurk in that region. But now, with the sound passed to the vault of an empty cavity, its curved extremity taking a backward direction, and its tip distinctly felt by the finger in the rectum, the diagnosis of a retroverted womb occupying the sacral excavation was quite readily established. The sound was also made to occupy a seemingly necessary place in determining the size of the womb—*i. e.*, whether above the normal average, or not; the relative length of the cervical and corporeal cavities, likewise the total length of both; the sensitiveness of the viscus; the thickness of its walls; its relations to other pelvic viscera; and finally, *inter alia*, it was employed as a repositor in correcting abnormal positions of the organ. Furthermore, one of its principal uses of late years has been in making applications of various medicines to the endometrium, in the treatment of many morbid conditions supposed to require medication by means of its use as an applicator.

This hasty and imperfect sketch of the "rise and progress" of the sound, has been made as preliminary to some criticisms, which it is the particular purpose of this paper to offer, upon its too indiscriminate and careless employment in the management of the diseases of women—errors presumed to be of constant occurrence.

It is believed by the writer, and by a goodly number of others who have both written and spoken on the subject, as well as by still others who have, as yet, remained silent thereon, which latter fact is learned by conversations with men of experience and judgment in regard to the uses of the sound, that younger physicians are particularly prone to resort to its use unnecessarily, and even in cases where it ought not to be employed at all, to the infinite, not to say sometimes irreparable, damage of the genital tract or health of woman.

Nor is this animadversion exclusively or alone applicable to the professional novitiate; for, sadly enough, it is a censure from which many of the more advanced practisers of the art of medicine cannot wholly or entirely escape. I, myself, have observed—and I dare say it is within the experience of many here present to affirm the like—a man of that large experience which pertains to years of practice, boldly invade the uterine cavity with the sound, twisting it hither and yon with a force and careless abandon to its possible consequences, that not only utterly amazed me at the time, but which, on reflection afterwards, brought a shudder of horror and a dismayful foreboding as to the resultant mischief which had already been produced, or which it were likely to cause.

To prevent misapprehension, and to escape, likewise, the possible charge of having assumed what might seem, on first thought, a self-asserted superiority of knowledge on this point, or of appearing to possess a "holier-than-thou" sort of wisdom thereon, permit me to declare that I do not, by any manner of means, hold myself to be wholly blameless concerning the improper use of the sound. On the contrary, I take unto myself a full share of the censure which I am venturing at this time, to also lay at the doors of others. Looking backwards upon the earlier years of my gynæcological work, I recall numerous instances in which I very much regret its employment at all, and still other, and much more numerous ones where I greatly deplore the *methods* of its use. These experiences, though unfortunate for such patients as have been the victims of my temerity, have, nevertheless, been laden with a golden freightage for me. I do not remember to have inflicted, in any case, a fatal, nor even an irrecoverable injury; but I have seen, as the work of my own hand, very serious threatenings in both these directions, and the lessons I have thereby learned, teach me to be ever fearful when taking into it the dreadful and ever-to-be-dreaded uterine sound. They have, moreover, taught me, even in those cases which seem most to invite its aid for diagnostic purposes, to use it with the utmost caution, circumspection, and gentleness.

It is the sum of these warnings, derived from such bitter experiences, which prompts me to speak out in terms of admonition, and to utter my feeble protest against the use of the sound, excepting in the plainest and most favorable conditions; and then, only, when other and better means of establishing a diagnosis, have failed to bring that exactitude which may be demanded in cases of great importance.

It would manifestly be difficult for one, in these days of improved methods of training, to mark out any definite line of proceedure that ought to be invariably adopted, with reference to any class of cases which might be presented for study or treatment; and, moreover, it should be considered particularly reprehensible to interdict or condemn, by reason of prejudice, any medicine or instrument that possesses any intrinsic merit, or which has any capabilities for good, whatsoever, lurking within itself when timely administered or intelligently employed. But I would most strenuously insist, in any case over which I could exercise control, that this particular instrument should only be appealed to as a final diagnostic resort in a juncture of extreme doubt. As for myself, I have, of late years, so seldom used the sound that I should be almost ashamed to exhibit those which I possess, they having grown so untidy—nay, almost rusty—with desuetude.

These are, indeed, but generalities, and I fear me and you are already exclaiming within ourselves, "Come to particulars, man!"

Fortunately, as far as meeting this demand is required, but unfortunately for those most concerned, the particulars are not few, nor the results obscure, upon which our indictment is brought.

Have we not repeatedly seen the endometrium, already so sensitive as to repel even the lightest touch, spring into violent inflammation as a result of contact with the uterine sound? Metritis—both peri- and para- —salpingitis, ovaritis, pelvic cellulitis, *et id genus omne*, are among the more direct results that have followed so closely upon its employment as to leave no reasonable doubt concerning cause and effect. These are formidable disturbances, in and of themselves, to deal with during the stage we are accustomed to call acute. In their more remote bearings upon the health of woman, they are far-reaching and extreme. How seldom does a woman recover from the immediate onslaught of one or other of these, and still other maladies, which may be directly chargeable to the use of the uterine sound, without a permanent—permanent unless fortunately relieved by art—impairment of her sexual functions in some important particular.

It is now sterility; then retroversion, or other displacement; and, again, hyperæsthesia of the genital tract, constituting one form of what Robert Barnes has so tritely named dyspareunia. Still, again, do we find a hyperplastic thickening of the uterine walls, with all its attendant phenomena, including uterine catarrh, than which there is no more obstinate form of so-called leucorrhœa; or, perhaps there is thickening, and consequent shortening of one or other of the broad ligaments, distorting and drawing out of place the uterine body, and rendering futile, until overcome, any successful attempts towards reposition of the displaced organ.

And yet again, may we have the pelvic inflammation resulting in abscesses of the connective tissue, which, in themselves, constitute some of the most formidable, not to say dangerous, of all the maladies liable to invade the pelvic cavity of woman. Still, and yet again, were it possible to group together, in a single table, the numberless instances where, innocently enough, to be sure, but none the less disastrously, women have been caused to abort, from the unwarrantable employment of the sound in the management of their special diseases, it is quite possible that the figures would be so appalling as to seem almost apochryphal.

Finally, though by no means of the last moment, may come those indescribable symptoms, remote, reflex and sympathetic in character, which we call neuroses—hystero-neuroses, if you please—that are as difficult to treat as they are to locate, but which, nevertheless, serve to make a woman more miserable, and are oftentimes more painful to witness, than the very agony of disease itself. These, together with the various neuralgias—ovarian, uterine, cystic and rectal—besides the backaches, spineaches and headaches, along with their associated functional disturbances, create a picture of woe and suffering, and sorrow altogether pitiable to behold, as well as conditions most obstinate to relieve.

I am aware, gentlemen, that the list is a long one, and the contemplation thereof not pleasant; but there is not, I affirm, a single one of the number which may not have its origin, directly or indirectly, in the unbecoming, unnecessary, or uncareful use of the uterine sound.

Mr. Lawson Tait, in a paper presented at the late meeting of the Medical Society of the State of New York, entitled "Methods of Diagnosis," discoursed at considerable length in regard to the uses of this instrument. Among other things, he said: "In the gynæcology of twenty years ago, which was pretty much the period at which the great master of the art left it, there still remained a survival of the battles which raged for many years concerning the speculum and the sound. The school of French gynæcology was charged with an altogether improper, and indeed, as it was urged, a very indecent frequency in the use of the speculum. On the other hand, the English school, with Simpson at its head, was fully as often and as loudly charged with an improper use of the sound.

The conclusion I have come to concerning the both of these instruments and both of these disputes is, that both sides were right and both were wrong.

"It is perfectly impossible for any novice in the diseases of women, to obtain an accurate notion as to the condition of the vaginal mucous surface of the os and cervix, and to some extent of the interior of the uterine canal, without the constant, I would almost say, invariable use of the speculum. It is also quite as impossible for that novice to form any notion as to the position of the fundus, or the relations of the uterus with the pelvic tumour, without the employment of the sound. But no practitioner can possibly be regarded as an accomplished specialist, at least by me, who uses one or the other of these instruments with great frequency. I have found in my own practice, that just as my experience increased so did both of them become unnecessary, until, concerning the speculum, it is a fact, that unless I want to do some operation, or make some special investigation within or beyond the vaginal cavity, the speculum is never employed at all; and for the discovery of the position of the uterus and its relations, the sound has almost ceased to be an advantage."

Again: "It is perfectly impossible for me to convey by any kind of description how I can tell by the touch, an inflamed vaginal mucous surface from one that is healthy; neither can I describe the feeling that the everted surface of the cervix gives to me, which declares the condition of chronic endometritis. But I know that my educated finger-tips can make this distinction. If, on the other hand, I discover a pelvic tumor, long pratice enables me to tell with almost perfect certainty, and without the use of the sound, that is a retroverted fundus, or adherent tube or ovary, or by its fading away toward the broad ligament, on one aspect of the uterus or another, that it is an intra-peritoneal hæmatocele; while the peculiar resistance of a myoma conveys to my mind an accurate impression which needs no probing of the uterus to substantiate. So a cyst reveals itself in a way that I cannot communicate. As a result of all this I very rarely use the sound. As a matter of fact, I have found that these two instruments, the speculum and the sound, as methods of diagnosis, have been productive of uniformly more harm than good."

I have made this, peradventure, all too lengthy quotation from Mr. Tait's paper, not for the purpose of endorsing it as a whole—for I most deferentially, but decidedly, dissent from that portion which pertains to the speculum — but simply with the design of showing what a master may do with his educated finger-tips ; and, still further, to show how unnecessary to him may become many of those instruments that he formerly regarded as essential, after he has taught his finger-ends such knowledge.

Not every man who practices the gynæcological art can expect to become a Tait — it were not well, probably, for many to become, in all respects, such as he — but all may do something towards improving their tactile sensitiveness ; the attempt, at least, should be made, for just in proportion as its acuteness is developed will a man's efficiency as a clinician strengthen, and correspondingly will his judgment, in doubtful or obscure cases, increase in weight and value.

The sound is nothing less nor more than a prolonged finger, and may therefore properly be dispensed with whenever and wherever the finger itself can be made available, provided it furnishes with accuracy the information sought. It certainly should never be used with any force whatever, its own weight being sufficient to carry it to any place where it can be made of any practical service. Furthermore, the os uteri should always be patulous to even a marked degree whenever it is employed, and the endometrium should likewise be free from any undue tenderness when touched by the sound.

Moreover, when it becomes necessary, from any cause, to appeal to such an instrument, the delicate virgin-silver probe of Sims is a much better and safer one than the Simpson sound, and even this ought never to be passed during a first interview if it were possible to avoid it, nor until one has become quite familiar with the topography, and all peculiarities of the sexual tract of his patient; nor, further, until it is first wrapped with film of absorbent cotton, to serve as a cushion to the metal.

Against the employment of the uterine sound as a repositor in cases of retroversion, it were scarcely possible to speak in too strong terms. If it is capable of doing harm when simply passed for diagnostic purposes, how much greater must be the danger of setting up formidable inflammatory conditions of the pelvic organs, if it were also employed to pry or rotate the retro-displaced womb into its normal position.

Whoever will make himself familiar with the comparative ease and absolute safety, with which a retroverted uterus can be unshipped from its moorings in the sacral excavation and caused to float in the cavity of the abdomen, through the intervention of the genu-pectoral posture, and the facilities for manipulation which it affords, will, I am very sure, never resort to the barbarous practice of employing any instrument within the uterine cavity, for the purpose of accomplishing this end. There is, I asseverate, no posterior displacement of the womb which is capable of being restored by any method, or which requires replacement at all, that may not be so replaced by intelligent, and, if need be, persistent manipulation with the patient in the genu-prone attitude.

In conclusion permit me to say, that from an experience and observation extending through a period of some years, and made up from a goodly number and variety of cases, I am prepared to corroborate Mr. Tait's remarks, just quoted, concerning the present inutility of the sound. It has played a good part in teaching us how to use our fingers to a much better advantage than was possible before its day; but it can now, with propriety, be laid aside in much of the gynæcological work, where it was formerly deemed necessary. The importance of training the finger-tips to a high state of tactile erudition cannot well be over-estimated, and, with its approach to that degree of perfection, which has for Mr. Tait a conspicuous exemplar, the necessity for the frequent employment of the sound will gradually cease; until finally, let it be hoped, we shall thus soon be rid of much of the opprobium which the young and growing art of gynæcology is now compelled to suffer.

284 Franklin Street.

